# Parcel-guided rTMS for depression

**DOI:** 10.1038/s41398-020-00970-8

**Published:** 2020-08-12

**Authors:** M. Moreno-Ortega, A. Kangarlu, S. Lee, T. Perera, J. Kangarlu, T. Palomo, M. F. Glasser, D. C. Javitt

**Affiliations:** 1 Division of Experimental Therapeutics, Department of Psychiatry, New York State Psychiatric Institute/Columbia University Medical Center, New York, NY USA; 2 Centro de Investigacion Biomedica en Red de Salud Mental (CIBERSAM), Madrid, Spain; 3 Department of Psychiatry, Radiology and Biomedical Engineering, Columbia University, New York, NY USA; 4 Department of Psychiatry and Biostatistics, New York State Psychiatric Institute/Columbia University, New York, NY USA; 5 Contemporary Care, Greenwich, CT USA; 6 State University of New York (SUNY) Upstate Medical University, Syracuse, NY USA; 7 Department of Psychiatry, Complutense University, Madrid, Spain; 8 Departments of Radiology and Neuroscience, Washington University Medical School, St. Louis, USA

**Keywords:** Depression, Scientific community

## Abstract

Transcranial magnetic stimulation (TMS) is an approved intervention for treatment-resistant depression (TRD), but current targeting approaches are only partially successful. Our objectives were (1) to examine the feasibility of MRI-guided TMS in the clinical setting using a recently published surface-based, multimodal parcellation in patients with TRD who failed standard TMS (sdTMS); (2) to examine the neurobiological mechanisms and clinical outcomes underlying MRI-guided TMS compared to that of sdTMS. We used parcel-guided TMS (pgTMS) to target the left dorsolateral prefrontal cortex parcel 46. Resting-state functional connectivity (rsfc) was assessed between parcel 46 and predefined nodes within the default mode and visual networks, following both pgTMS and sdTMS. All patients (*n* = 10) who had previously failed sdTMS responded to pgTMS. Alterations in rsfc between frontal, default mode, and visual networks differed significantly over time between groups. Improvements in symptoms correlated with alterations in rsfc within each treatment group. The outcome of our study supports the feasibility of pgTMS within the clinical setting. Future prospective, double-blind studies of pgTMS vs. sdTMS appear warranted.

## Introduction

Transcranial magnetic stimulation (TMS) over left dorsolateral prefrontal cortex (L-DLPFC) is an FDA-approved treatment for treatment-refractory depression (TRD), but only partially effective, with response and remission rates of 41.2% and 35.3%, respectively^1,2^. Current targeting techniques for rTMS rely on distance from motor cortex (“5-cm rule”). This approach provides only approximate targeting of L-DLPFC, with no consistent differentiation among potentially relevant DLPFC subregions. A long-term goal of TMS research is to guide TMS targeting on a personalized basis, in order to improve consistency of targeting across individuals. Nevertheless, optimal methods for targeting remain to be developed^3–5^.

Recently, we evaluated resting-state functional connectivity (rsfc) changes pre/post electroconvulsive therapy (ECT)^6^, in order to identify specific rTMS targets within L-DLPFC. Moreover, we applied a prespecified surface-based multimodal parcellation scheme that divides DLPFC into 13 distinct subregions (“parcels”), in order to permit consistent identification across individuals (Supplementary Fig. 1). We observed greatest correlation of ECT response to changes in rsfc involving two specific DLPFC parcels—46 and p9-46v—on one part, and brain regions considered to be involved in the pathophysiology of depression, including the anterior default mode network (DMN, s32) and ventral visual region (VIS, ventral) on the other.

Here, we evaluated effects of excitatory (10 Hz) “parcel-guided” rTMS (pgTMS) targeted at DLPFC(46) using individualized MRI, relative to effects of standard “5-cm rule” rTMS (sdTMS). In addition to symptoms, we evaluated rsfc changes induced by pgTMS vs. sdTMS, relative to patterns of change previously observed pre/post ECT. We hypothesized that pgTMS would show beneficial effects in TRD individuals who were nonresponsive to sdTMS, and that these changes would be associated with differential effects on underlying network connectivities^6^.

## Methods

### Participants

We obtained Hamilton Depression Rating Scale (HDRS) and resting-state functional MRI (rsfMRI) scans pre/post rTMS in a group of ten TRD subjects who were nonresponders to sdTMS and who received pgTMS targeted at parcel 46, and compared both magnitude of change and rsfc correlates relative to a group of 22 rTMS-naive TRD subjects given sdTMS (Supplementary Fig. 2). All subjects met DSM-IV criteria for a major depressive episode according to the diagnostic assessment by the Structured Clinical Interview Patient Edition.

The ten subjects studied pre–post pgTMS were drawn from a pool of clinical patients at different locations, who did not respond adequately to standard ongoing treatments. All pgTMS treatments were done at the Greenwich location (ages 18–60). All subjects were right-handed and without severe medical conditions. The NYSPI/CUMC and Western Institutional Review Board approved this study, which was registered online (clinicaltrials.gov registration NCT02974296). All participants provided written informed consent.

### Treatment

All treatments were open label and administered using a NeuroStar Therapy System. sdTMS used standard “5-cm rule” targeting. pgTMS stimulation locations were determined using a Brainsight (Rogue Research, Montreal, Canada) neuronavigation system. All patients completed 36 sessions, with 5×/week for 6 weeks, followed by 3 weeks taper off. TMS was administered at 120% of motor threshold at a frequency of 10 Hz, for a total of 3000 pulses per session. To obtain preliminary data on differences between standard vs. individualized targeting, the same TMS device and treatment protocol were used in the sdTMS group (as part of routine clinical care) and the pgTMS group. After completion of TMS treatments patients received a second rsfMRI scanning followed by the HDRS-24.

### MRI

Anatomical images and rsfMRI were collected using a GE Discovery MR750 3.0 Tesla full-body MRI. DLPFC, DMN, and VIS regions, were operationalized respectively as parcels 46, s32, and the VIS ventral region^7^. Target location for pgTMS was determined using vertices within DLPFC(46) that showed greatest anticorrelation to s32 (Supplementary Fig. 3b).

### Statistics

Preplanned analyses focused on mixed effect regression models to test whether change in rsfc pre/post both sdTMS and pgTMS differs by treatment group. Association between change in HDRS and that of each rsfc was measured using linear regression. Group difference in the association was tested by adding the interaction term between change in rsfc and treatment group. Fisher’s *z-*transform was applied to individual rsfc maps before group level analyses; corrected *p*-values using FDR^8^ controlled multiple comparison correction were computed (see Supplementary Methods for further details). Bartlett’s test was performed to test variance homogeneity across groups. Wherever equal variance hypothesis was rejected, we refit the model allowing different variance by group.

## Results

### Symptoms

**Fig. 1:**
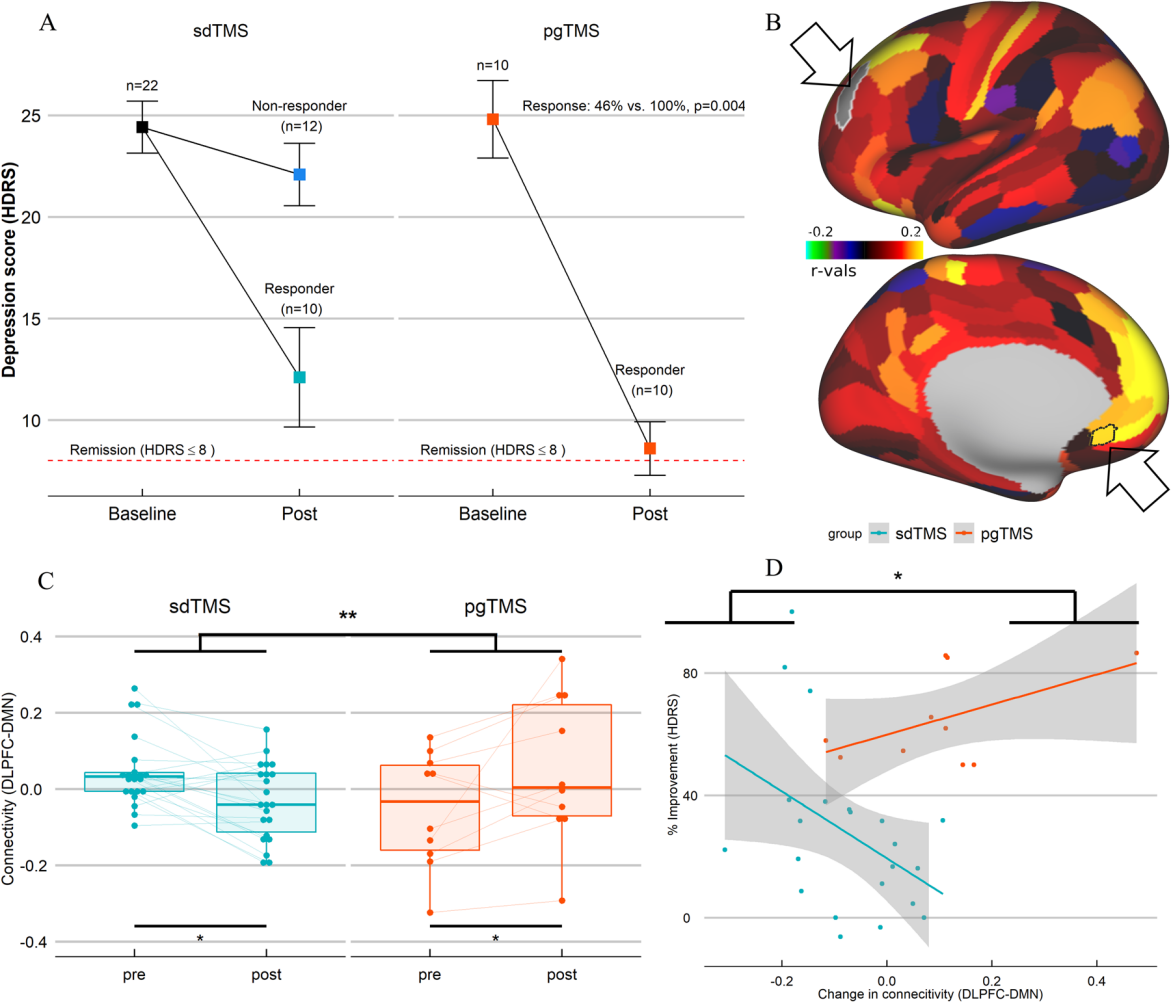
Response rate and change between DLPFC(46) and DMN(s32) connectivity. **a** Difference in response rate between groups (sdTMS vs. pgTMS). **b** Brain images display the contrast pgTMS vs. sdTMS of the parcellation-based connectome between left DLPFC(46) (lateral view) used as seed and left DMN(s32) (medial view), both highlighted with black arrows; colors represent parcels with increased negative (blue violet) or increased positive (red yellow) correlation with left DLPFC(46) in pgTMS vs. sdTMS. **c** Bar plot with rsfc change difference between left DLPFC(46) and left DMN(s32) by group, and of each group. **d** Correlation plot with group difference in the association between change in HDRS and change in rsfc. Corrected *p*-values using FDR^8^ controlled multiple comparison correction are displayed.

Of the ten patients treated with pgTMS following nonresponse to sdTMS, all (100%) showed a significant response, with 50% showing full remission. By contrast, 46% of patients treated with sdTMS (10 out of 22) showed a significant response, with 18% remission. The difference in mean symptoms change (64 ± 15% vs. 28 ± 27%, *t* = −3.99, *p* < 0.001) and response rate (*χ*² = 6.56, *p* = 0.004) between groups was statistically reliable (Fig. 1a).

### rsfc

**Fig. 2:**
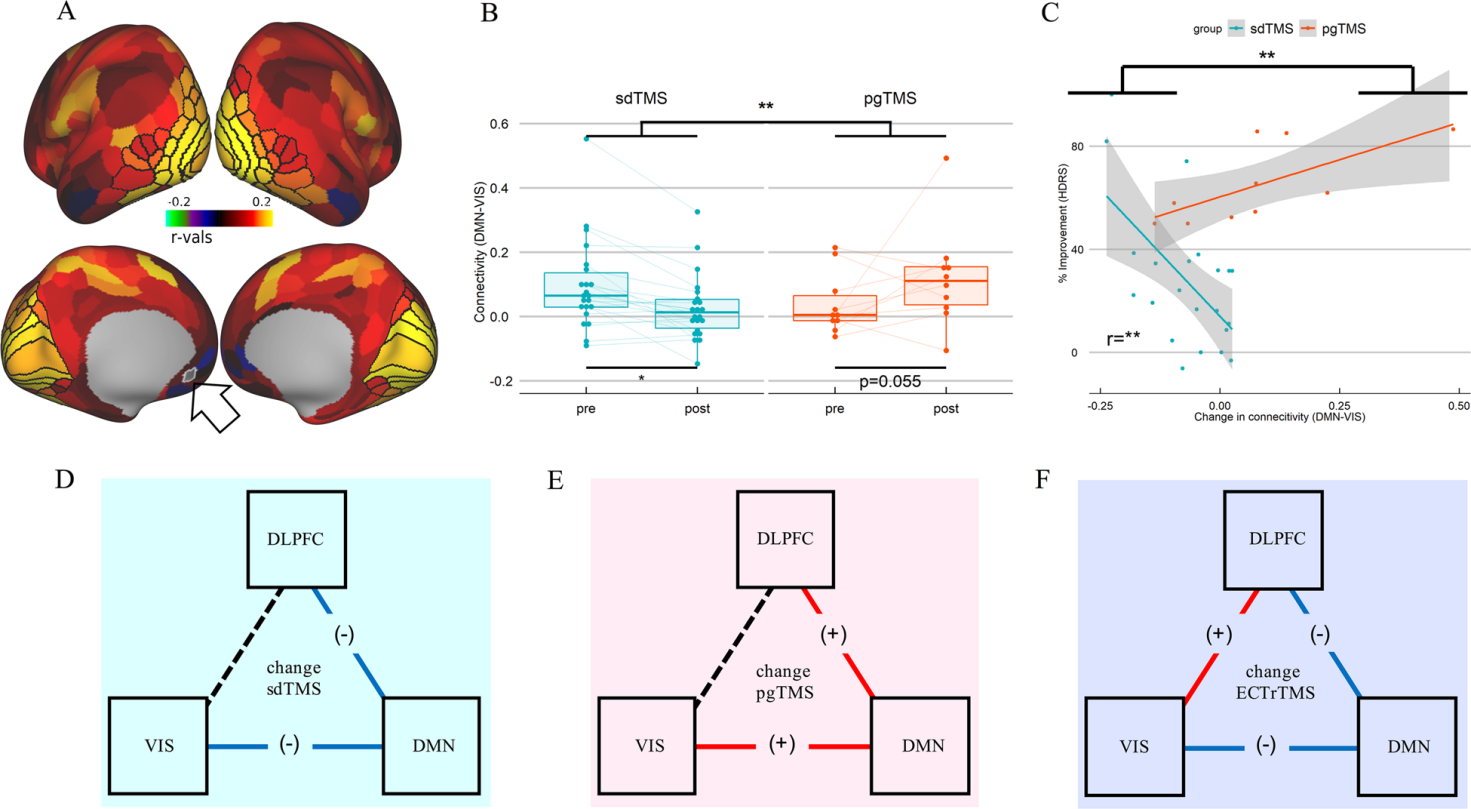
Change between DMN(s32) and VIS(ventral) connectivity. **a** Brain images display the contrast pgTMS vs. sdTMS of the parcellation-based connectome between left DMN(s32) (medial view) used as seed (highlighted with a black arrow) and bilateral VIS(ventral) (lateral and medial views), all parcels within the ventral region are highlighted in black; colors represent parcels with increased negative (blue violet) or increased positive (red yellow) correlation with left DMN(s32) in pgTMS vs. sdTMS. **b** Bar plot with rsfc change difference between left DMN(s32) and bilateral VIS(ventral) by group, and of each group. **c** Correlation plot with group difference in the association between change in HDRS and change in rsfc. Corrected *p*-values using FDR^8^ controlled multiple comparison correction are displayed. (**d**–**f**) Schematic representation of rsfc structure after sdTMS (**d**), pgTMS (**e**), or ECT (**f**); colored straight lines show significant connections, with positive (+) or negative (−) rsfc correlation; black dot lines show absence of significant connections.

No baseline differences in rsfc were found between the groups (Supplementary Table 1). Connectivity between DLPFC(46) and DMN(s32) (Fig. 1b), or between DMN(s32) and VIS(ventral) (Fig. 2a) differed significantly over time between groups in post–pre changes (Figs. 1c and 2b, Supplementary Table 2). There were also differences in connectivity by group (Supplementary Table 3). We found increased negative correlation between DLPFC(46) and DMN(s32) (Fig. 1c), and between DMN(s32) and VIS(ventral) (Fig. 2b), following sdTMS.

By contrast, increased positive correlation between DLPFC(46) and DMN(s32) (Fig. 1c), and between DMN(s32) and VIS(ventral) (Fig. 2b) followed pgTMS. The correlation between change in depression scores (HDRS) and rsFC also differed by group (Supplementary Table 4.1). Specifically, individuals in the sdTMS group showed greater negative connectivity associated with improvement in depression scores (Figs. 1d and 2c, Supplementary Table 4.2), with a pattern similar to that previously observed pre/post ECT treatment^6^ (Fig. 2d, f), while the opposite pattern was found in the pgTMS (Fig. 2e). Results stayed very similarly (Supplementary Table 5.1–5.4) after potential outliers were removed, and still significant after controlling for multiple comparison correction.

Similar results were obtained in both groups for other VIS regions, including dorsal, MTC, and early (Supplementary Results). Moreover, results remained significant following multiple comparison correction.

### Relative location

For all sdTMS subjects, we approximated the likely target location by plotting the MNI coordinates −41, 16, and 54 of the average 5-cm rule^9^ unto the Glasser parcellation scheme^10^. Targets mapped primarily to area 8Av (Supplementary Fig. 3a), known to be part of the DMN. Responders and nonresponders to sdTMS were not notably different in estimated target location. For pgTMS subjects, targets fell within the most anterior/ventral region of DLPFC(46) (Supplementary Fig. 3b); known to be part of the fronto-parietal network.

## Discussion

Limitations of the “5-cm rule” for guiding sdTMS are well understood^5^. Nevertheless, no alternative targeting strategies have attracted widespread use. Here, we take advantage of recent advances in surface-based multimodal parcellation of cortex^10^, as well as our recent findings of pre/post changes in rsfc following successful ECT^6^ to refine targeting for rTMS in depression. As predicted, we observed significant improvement following pgTMS in individuals who were nonresponsive to sdTMS. Moreover, we observed opposite patterns of rsfc change following pgTMS vs. sdTMS, reinforcing the importance of precise target localization for rTMS stimulation.

DLPFC(46) falls broadly within a region that is anticorrelated with subgenual anterior cingulate cortex (sgACC) (Supplementary Fig. 1b). Our findings, therefore, are broadly consistent with a large body of work suggesting that optimal targets for rTMS fall broadly within sgACC “anticorrelated” region of L-DLPFC^9,11^. Nevertheless, operationalizing this approach using personalized rsfc measures has proven challenging, in part because of long acquisition times needed to get stable, single-subject rsfc solutions^3,12–14^.

Here, we demonstrate that utilization of a surface-based parcellation scheme gives unexpectedly superior results vs. sdTMS. In our recent ECT study, we observed greater connectivity changes in a circuit involving L-DLPFC(46), s32, and VIS cortex than between L-DLPFC(46) and sgACC(25), suggesting potentially greater importance of these connections vs. the more traditionally studied connections to sgACC. Our present pre/post results further support the importance of these connections. However, we cannot discount the potential role of sgACC in the present study, since the surface-based parcellation used for analyses separates areas 25 and s32 (or subgenual 32), a difference that might be lost in voxel-based definitions of sgACC (or BA25). Our findings also suggest that s32 may serve as an appropriate target for deep brain stimulation, along with area 25.

In our study, all subjects first received the FDA-approved “5-cm rule” treatment (sdTMS) before being entered into experimental treatment. In our group of ten subjects who did not respond to sdTMS, all subjects who received pgTMS responded to treatment as defined based on ≥25% reduction in symptoms, and 50% (5/10) obtained remission. Both mean symptom change and % of subjects showing treatment response were significantly greater for pgTMS than for a parallel group of subjects, who received sdTMS for the first time, as well as those expected from rTMS meta-analyses^15^.

In addition to symptom change, we also observed rsfc changes within the predefined DLPFC, DMN, and VIS circuit, suggesting objective physiological effects. Surprisingly, we observed an opposite pattern of rsfc change in the sdTMS and pgTMS groups. sdTMS produced changes similar to those observed pre/post ECT, albeit weaker, suggesting a convergent mechanism of effect. By contrast, pgTMS produced an opposite pattern of change.

Interactions between DLPFC(46), DMN(s32), and VIS(ventral) are critical for normal goal-directed VIS activity^16^, and is consistent with spatio-temporal dynamics of interactions between emotional stimulus and task-driven attention^17,18^. In particular, the simultaneous increase in DLPFC–DMN and DMN–VIS connectivity in the pgTMS group might underlie the mechanism by which frontal structures regulate attention and emotion influences on VIS cortex.

A mechanistic explanation of this interaction could be that both emotional salience feedback from the DMN and top-down signaling from DLPFC regions synergistically increase processing in the VIS cortex^17^. Long-range cortico–cortical projections might act through local microcircuits to exert spatially specific top-down modulation of sensory processing^19^. Restoration of these small local circuits might be crucial for the development of long-range cortico–cortical projections to exert specific top-down modulation of attention and emotion influences on sensory processing.

The present results suggest a dysfunctional pattern associated with depression that can be modulated bidirectionally (top-down/bottom-up), depending upon the precise location of the rTMS target. The present study reinforces first that small shifts in target location within DLPFC may produce large differences in outcome, and that alterations in rsfc patterns may be helpful both in selection of subjects and monitoring of response^20,21^.

The present study differs from prior studies that have used structurally^22^ or functionally^23–25^ guided rTMS in that it uses surface—rather than volume-guided targeting—and considers rsfc to DMN and VIS, as well as sgACC based upon our recent findings. Surface-based targeting provides approximately threefold greater spatial localization than does traditional volumetric targeting^26^. Moreover, use of a predesignated parcellation scheme greatly reduces data dimensionality and permits greater comparability of results across studies. In the present study, we further refined targeting on an individual basis using rsfc to DMN, which implicated ventral regions of area 46 (Supplementary Fig. 3b). Nevertheless, dispersion of the target was small across subjects, suggesting that targeting might be accomplished using structural MRI alone.

Patients receiving sdTMS in this study showed response and remission rates similar to those previously reported^5^. An unanswered question is whether the greater treatment response and higher remission rates with pgTMS relative to both, sdTMS subjects and prior literature, are because the subjects were specifically chosen based on nonresponsiveness to sdTMS, or whether similar effects might be observed even in unselected TRD patients. Also, because pgTMS was used as a continuation to sdTMS, patients in the pgTMS received longer total treatment than those in the sdTMS group. Prior continuation studies have suggested response rates of 11–40% with continued high-frequency stimulation to L-DLPFC^27,28^, but higher response rates following a change in either the target or the device^28,29^.

Cole et al.^25^ have recently published a similar study using a much more aggressive stimulation TMS protocol. Their study was also small and open label, and also showed extremely high response rates (>90%) in a sample of unselected TRD patients, including a subsample of nonresponders to TMS. However, some differences between the studies require further discussion. First, we used targeting based upon a structural atlas, rather than mapping rsFC patterns for each person. The use of a structural atlas reduces the computational burden needed to calculate rsFC patterns for individual subjects, and thus may be more clinically applicable. Second, we used a standard FDA-approved stimulation approach (10 Hz stimulation, 3000 pulses/session, 30 sessions) to better match ongoing TMS treatment. Nevertheless, our proposed targeting approach could potentially be used in future intensive stimulation studies.

The present study is limited by the small sample size, open design, and sequential treatment. Nevertheless, it demonstrates feasibility of the personalized surface and parcel-based approach for guiding TMS treatment, as well as the use of pre/post rsfc imaging to analyze underlying therapeutic mechanisms. Future parallel-group studies are needed to directly compare efficacy of pgTMS vs. sdTMS, as well as feasibility of the parcel-guided approach within larger treatment samples.

## Supplementary information

Supplementary documentation for: Parcel-guided rTMS for depression

Supplementary Table 1.

Supplementary Table 2.

Supplementary Table 3.

Supplementary Table 4.1.

Supplementary Table 4.2.

Supplementary Table 5.1.

Supplementary Table 5.2.

Supplementary Table 5.3.

Supplementary Table 5.4.

Supplementary Figure 1.

Supplementary Figure 2.

Supplementary Figure 3.
