# Supplementary material for: Parcel-guided rTMS for depression
Source: Transl Psychiatry. 2020 Aug 12;10:283. doi: 10.1038/s41398-020-00970-8 (PMC7423622; doi:10.1038/s41398-020-00970-8)
Supplement: Supplementary file 1 — Supplementary documentation for: Parcel-guided rTMS for depression [file 41398_2020_970_MOESM1_ESM.docx]

Supplementary documentation for: Parcel-guided rTMS for depression

Moreno-Ortega M*^1,2^, Kangarlu A^3^, Lee S^4^, Perera T^5^, Kangarlu J^6^, Palomo T^2,7^, Glasser MF^8^ and Javitt DC^1^.

Supplementary Methods

*Participants:* Participants were 32 patients with treatment resistant depression (TRD) recruited from Contemporary Care Clinics (ages 18-60), who met DSM-IV criteria for a Major Depressive Episode (MDE) according to the diagnostic assessment by the Structured Clinical Interview Patient Edition (SCID-P), with scores of 18 or greater (mean=24.5, SD=5.9) on the 24-Hamilton Depression Scale (HDRS-24). Patients were divided in two independent groups: (1) TRD patients prescribed for the first time to standard TMS (sdTMS) (n=22), or (2) non-responders to sdTMS who received parcel-guided TMS (pgTMS) (n=10). Patients with comorbid other Axis I or Axis II psychiatric disorders were excluded. All subjects were right-handed and without severe medical conditions. The NYSPI/CUMC and Western Institutional Review Board approved this study. All participants provided written informed consent. This study was performed in accordance with all relevant guidelines and regulations.

*Procedure:* Study procedures occurred over 9 weeks. First 6 weeks began with functional MRI scanning followed by the Hamilton[^1^](#_ENREF_1) rating scale (HDRS, 24 items). One week after the fMRI, patients started their sdTMS or pgTMS treatments. All patients completed 36 sessions, with 5tx/week for 6 weeks, followed by 3 weeks taper off. After completion of TMS treatments patients received a second functional MRI scanning followed by the HDRS-24. Individual DMN(left s32) rsfc maps from patients undergoing pgTMS were computed at baseline, vertices within DLPFC(left 46) that showed greatest anti-correlation with DMN(left s32) were selected as personal targets (peak detection using wb_command -cifti-extrema) (**Suppl. Fig. 3B**). Patients undergoing sdTMS were targeted following the 5-cm rule (**Suppl. Fig. 3A**). Parcellation-based connectomes were generated for each individual pre/post TMS treatments. Patients were classified as responders to TMS with at least 25% decrease in HDRS-24 scores from baseline, or as remitters with HDRS post-score of 8 or lower.

*TMS treatments:* Using the NeuroStar Therapy System, the subject’s motor threshold (MT) is determined and the coil placed over the left DLPFC. The "standard" procedure of coil positioning locates the left DLPFC at 5-cm anterior to the "hand motor hotspot" along the curvature of the scalp. Alternate stimulation site within the DLPFC in each patient was used to personalize treatments based on their RSFC MRI, therefore its application is considered off-label. Brainsight Neuronavigation device was used to determine the location of the coil on the subject’s head. While holding the coil on the head, Brainsight indicates in which direction to move the coil to quickly and accurately place the coil over the target. One placed, the system monitors the coil’s activity and can automatically record the coil’s position and orientation w.r.t. target for quality assurance. All patients received stimulation over the left DLPFC at 120% of the MT at a frequency of 10Hz; for a total of 3,000 pulses per session. A subset of sdTMS patients also received low frequency (1 Hz) stimulation to right DLPFC following the 5-cm rule.

*fMRI Data Acquisition and Processing:* High resolution anatomical images and resting-state functional MRI were collected pre/post TMS. Post study imaging was performed approximately 48-72 hours after the last treatment to allow sufficient time for recovery from treatment. Anatomical and functional imaging data from each subject were collected and processed using acquisition guidelines and processing pipelines provided by the Human Connectome Project (HCP)[^2^](#_ENREF_2). High-resolution functional imaging scans were carried out at Citigroup Biomedical Imaging Center (CBIC) of Weill-Cornel Medicine using a GE Discovery MR750 3.0 Tesla full body MR system equipped with a 32-channel phased array head coil (Nova Medical, Wilmington MA).

Subjects were placed in the scanner with head cushioning to restrict head movement. Following localizer scans (5 minutes), distortion correction scans (B0 fieldmap), a pair of T1-weighted images, and T2-weighted images are acquired over 25 minutes, followed by three 10-minute resting state fMRI scan. Total scan time was 55 min. Sessions were conducted prior to and following completion of the course of TMS. T1-weighted images acquired for anatomical co-registration were transverse T1-weighted BRAVO sequence with the following acquisition parameters: 3D sagittal, 0.8mm isotropic, matrix size=300x300, slices=220, TR=7.856ms, TE=3.108ms, flip angle=12°, TI=450ms and CUBE T2-weighted image was acquired with these parameters; 3D sagittal, 0.8mm isotropic, matrix=320x320, # of slices=220, TR=2500ms, TE=95.708 ms, flip angle=90°.

Functional images were acquired with a GE-EPI sequence (2mm isotopic, slice plane=transverse, MUX=6, TR=850ms, TE=25ms, matrix=96x96, slices=11, phase encode =A->P); an instruction was given to all patients before the functional MRI sequence to keep their eyes open. Images were transferred to a workstation with the HCP processing pipeline version 4 installed. The pipeline implements standard automated structural and fMRI processing (movement correction, atlas realignment, creation of cortical surface model etc.) with FSL 6, FreeSurfer v5.3.0-HCP, and Connectome Workbench v1.3.2, and two additional procedures that improve upon standard preprocessing: required use of top-ups sequences to “undistort” GE-EPI images and creation of a “gray-ordinate” CIFTI-format files that only contained data from cortical and subcortical gray matter. fMRI data was further processed (after Functional Preprocessing is complete) using the FMRIB group's ICA-based Xnoiseifer – FIX. This processing regressess out motion timeseries and artifact ICA components (ICA run using Melodic and components classified using FIX)[^3^](#_ENREF_3). The order of pipelines included PreFreeSurfer, FreeSurfer, PostFreeSurfer (using MSMSulc), fMRIVolume, fMRISurface, MR ICA+FIX, MSMAll (MSM Surface Registration) and DedriftAndResample (apply MSMAll) before resting state analysis[^2^](#_ENREF_2)^,^[^4^](#_ENREF_4).

*Definition of ROIs:* Regions of interest (ROI) for these analyses were defined using the multi-modal parcellation[^5^](#_ENREF_5) of human cortex. Areas within the left DMN (s32) and left DLPFC (46) were selected following our previous results on predictive models of ECT response. The VIS network has been divided into primary VIS early (V1-V4), MTC, dorsal and ventral regions. We first focused on the ventral VIS region, based on our previous results on ECT. We then extended our analyses on VIS regions to also cover early and the above mentioned VIS association regions, grouped into separate subsystems: early, MTC and dorsal[^5^](#_ENREF_5).

*RSFC analyses:* Using the HCP’s multi-modal parcellation[^5^](#_ENREF_5), the parcellation-based connectome was generated for each individual using Connectome Workbench v1.3.2 (using wb_command -cifti-parcellate and wb_command -cifti-correlation). Correlation values from left DLPFC(46) and left DMN(s32), and from left DLPFC(46) or left DMN(s32) and left/right VIS regions were extracted from each individual parcellation-based connectome. Connectivity measures between left 46 or left s32 and the left/right VIS parcels were previously averaged within each VIS region (early, MTC, dorsal and ventral) and across hemispheres. Analyses focused on whether rsfc change and the association with change in HDRS differed by group.

Fisher’s z transform was applied to individual parcellation-based connectome before group level analyses; uncorrected p-values were reported as only 3 comparisons were made; corrected p-values using FDR[^6^](#_ENREF_6) controlled multiple comparison correction were computed to see whether any of the uncorrected p-values survived.

*Statistics:* Before running the analysis, we checked whether there is any difference in HDRS and rsfc measures between the two treatment group to check potential confounding effects of the baseline status. Linear regression was conducted followed by multiple comparison correction controlling for false discovery rate (FDR)[^6^](#_ENREF_6).

To test whether change in rsfc differs by treatment group, mixed effect regression models were conducted with rsfc measures as dependent variable, time (two levels: pre, post treatment), group (two levels: sdTMS, pgTMS), and time by group interaction as fixed effects. Within-subject correlation was accounted by adding random intercept. The significant time by group interactions with multiple comparison corrected p-values less than 5% were considered as significant group difference. Post-hoc least-squared means of the post-pre change of each group were computed to see actual directions of each measures.

We tested the association between change in HDRS and that of each rsfc measure using linear regression with change in HDRS as a dependent variable, change in rsfc and group as independent variable. Group difference in the association was tested by adding the interaction term between change in rsfc and treatment group.

Change in rsfc scores greater than .4 were considered as outliers, although there is no evidence that these values are illegitimate. This was part of sensitivity analysis to make sure the findings were not driven by some outliers.

Supplementary Results

Connectivity between DMN(s32) and VIS(early) (t=3.199, p=0.003), VIS(MTC) (t=2.943, p=0.006) or VIS(dorsal) (t=2.937, p=0.006) differed significantly over time between groups in post-pre changes. There were also differences in DMN(s32) connectivity by group; i.e., VIS(early) (sdTMS: t=-3.013, p=0.005; pgTMS: t=1.827, p=0.076), VIS(MTC) (sdTMS: t=-2.880, p=0.007; pgTMS: t=1.608, p=0.117) or VIS(dorsal) ) (sdTMS: t=-3.076, p=0.004; pgTMS: t=1.468, p=0.151). As with VIS(ventral), increased negative correlation between DMN(s32) and VIS(early), VIS(MTC) or VIS(dorsal) followed sdTMS vs. pgTMS.

The correlation between change in depression scores (HDRS) and rsFC also differed by group; DMN(s32) and VIS(early) (t=2.622, p=0.014), VIS(MTC) (t=2.909, p=0.007) or VIS(dorsal) (t=2.471, p=0.020). Specifically, individuals in the sdTMS group showed greater negative connectivity associated with improvement in depression scores, while the opposite pattern was found in the pgTMS.

Supplementary References

1 Williams, J. B. A structured interview guide for the Hamilton Depression Rating Scale. *Arch Gen Psychiatry* **45**, 742-747 (1988).

2 Glasser, M. F. *et al.* The minimal preprocessing pipelines for the Human Connectome Project. *NeuroImage* **80**, 105-124, doi:10.1016/j.neuroimage.2013.04.127 (2013).

3 Salimi-Khorshidi, G. *et al.* Automatic denoising of functional MRI data: combining independent component analysis and hierarchical fusion of classifiers. *NeuroImage* **90**, 449-468, doi:10.1016/j.neuroimage.2013.11.046 (2014).

4 Robinson, E. C. *et al.* Multimodal surface matching with higher-order smoothness constraints. *NeuroImage* **167**, 453-465, doi:10.1016/j.neuroimage.2017.10.037 (2018).

5 Glasser, M. F. *et al.* A multi-modal parcellation of human cerebral cortex. *Nature* **536**, 171-178, doi:10.1038/nature18933 (2016).

6 Benjamini, Y., and Hochberg, Y. Controlling the false discovery rate: a practical and powerful approach to multiple testing. *Journal of the Royal Statistical Society Series B (Methodological)* **57**, 289-300 (1995).

Supplementary Figures

**Supplementary Figure 1. DLPFC_pos_ *versus* DLPFC_neg_.** SgACC RSFC map from an independent cohort of 1200 healthy subjects from HCP was generated to identify more correlated (DLPFC_pos_) (A) and anticorrelated (DLPFC_neg_) (B) left DLPFC coordinates in the same sample of healthy subjects; colors represent surface vertices with negative (blue-violet) or positive (red-yellow) correlation with the sgACC (area 25).

**Supplementary Figure 2.** **Flow diagram with study procedures according to CONSORT statement.**

**Supplementary Figure 3. DLPFC targets for TMS.** Brain images display the baseline s32 RSFC map from sdTMS (A) *versus* pgTMS (B) to illustrate differences in target location by plotting MNI coordinates -41, 16, 54 of the average 5-cm rule within the DLPFC(8Av) (A) *versus* vertices within the DLPFC(46) that showed greatest anti-correlation to s32 (B); colors represent surface vertices with negative (blue-violet) or positive (red-yellow) correlation with s32.

Supplementary Tables

**Supplementary Table 1.** Baseline treatment group difference in the rsfc measures.

**Supplementary Table 2.** Group x Time interaction results to test rsfc change difference by group.

**Supplementary Table 3.** LSMEANS to test post-pre change by group.

**Supplementary Table 4.1.** Test whether association between change in HDRS and change in RSFC differ by group.

**Supplementary Table 4.2.** Test association between change in HDRS and change in RSFC by group.

**Supplementary Table 5.1.** Group x Time interaction after outlier removed.

**Supplementary Table 5.2.** LSMEANS to test post-pre change by group after outlier removed.

**Supplementary Table 5.3.** Test whether association between change in HDRS and change in RSFC differ by group after outlier removed.

**Supplementary Table 5.4.** Test association between change in HDRS and change in RSFC by group after outlier removed.
