# Supplementary material for: Parcel-guided rTMS for depression
Source: Transl Psychiatry. 2020 Aug 12;10:283. doi: 10.1038/s41398-020-00970-8 (PMC7423622; doi:10.1038/s41398-020-00970-8)
Supplement: Supplementary file 2 — Supplementary Table 1. [file 41398_2020_970_MOESM2_ESM.docx]

|  | **sdTMS** | **pgTMS** | **t** | **p** | **Corrected p-value** |  | **Equal variance**  **(corrected p)** |
| --- | --- | --- | --- | --- | --- | --- | --- |
| **46 to s32** |  |  |  |  |  |  |  |
| Mean (SD) | 0.0437 (0.0917) | -0.0541 (0.151) | -2.273 | 0.030 | 0.090 |  | 0.204 |
| **46 to ventral** |  |  |  |  |  |  |  |
| Mean (SD) | 0.123 (0.138) | 0.132 (0.0976) | 0.197 | 0.845 | 0.845 |  | 0.773 |
| **s32 to ventral** |  |  |  |  |  |  |  |
| Mean (SD) | 0.0960 (0.141) | 0.0387 (0.0953) | -1.167 | 0.252 | 0.378 |  | 0.607 |
