# Supplementary material for: Parcel-guided rTMS for depression
Source: Transl Psychiatry. 2020 Aug 12;10:283. doi: 10.1038/s41398-020-00970-8 (PMC7423622; doi:10.1038/s41398-020-00970-8)
Supplement: Supplementary file 3 — Supplementary Table 2. [file 41398_2020_970_MOESM3_ESM.docx]

|  | **Estimate** | **Std..Error** | **df** | **t.value** | **p-value** | **sig** | **corrected.p** |  | **Equal variance corrected p^1^** |
| --- | --- | --- | --- | --- | --- | --- | --- | --- | --- |
| **46 to s32** | 0.180 | 0.047 | 33.972 | 3.836 | 0.001 | *** | 0.002 | ** | 0.402 |
| **46 to ventral** | 0.014 | 0.059 | 34.474 | 0.238 | 0.814 |  | 0.814 |  | 1.000 |
| **s32 to ventral** | 0.150 | 0.044 | 33.374 | 3.374 | 0.002 | ** | 0.003 | ** | 0.011 |
| **s32 to ventral**^2^ | 0.150 | 0.045 | 30.000 | 3.264 | 0.003 | ** |  |  |  |

^1^ Bartlett’s test on the pre-post change score were performed.

^2^ The variable was reanalyzed after allowing different variance by group and the results remained almost identical.
