# Supplementary material for: Parcel-guided rTMS for depression
Source: Transl Psychiatry. 2020 Aug 12;10:283. doi: 10.1038/s41398-020-00970-8 (PMC7423622; doi:10.1038/s41398-020-00970-8)
Supplement: Supplementary file 4 — Supplementary Table 3. [file 41398_2020_970_MOESM4_ESM.docx]

|  | **group** | **estimate** | **SE** | **df** | **t.ratio** | **p.value** | **sig** | **corrected.p** | **sig.corrected** |
| --- | --- | --- | --- | --- | --- | --- | --- | --- | --- |
| **46 to s32** | sdTMS | -0.076 | 0.026 | 34.257 | -2.915 | 0.006 | ** | 0.024 | * |
| **46 to s32** | pgTMS | 0.103 | 0.039 | 34.257 | 2.661 | 0.012 | * | 0.024 | * |
| **46 to ventral** | sdTMS | 0.040 | 0.033 | 34.626 | 1.189 | 0.243 |  | 0.284 |  |
| **46 to ventral** | pgTMS | 0.054 | 0.049 | 34.626 | 1.088 | 0.284 |  | 0.284 |  |
| **s32 to ventral** | sdTMS | -0.070 | 0.025 | 34.183 | -2.816 | 0.008 | ** | 0.024 | * |
| **s32 to ventral** | pgTMS | 0.080 | 0.037 | 34.183 | 2.171 | 0.037 | * | 0.055 |  |
