# Supplementary material for: Parcel-guided rTMS for depression
Source: Transl Psychiatry. 2020 Aug 12;10:283. doi: 10.1038/s41398-020-00970-8 (PMC7423622; doi:10.1038/s41398-020-00970-8)
Supplement: Supplementary file 5 — Supplementary Table 4.1. [file 41398_2020_970_MOESM5_ESM.docx]

|  | **Estimate** | **Std..Error** | **t.value** | **Pr…t..** | **sig** | **corrected.p** | **sig.corrected** |
| --- | --- | --- | --- | --- | --- | --- | --- |
| **46 to s32** | 158.519 | 66.021 | 2.401 | 0.023 | * | 0.035 | * |
| **46 to ventral** | 74.335 | 65.455 | 1.136 | 0.266 |  | 0.266 |  |
| **s32 to ventral** | 254.373 | 65.121 | 3.906 | 0.001 | *** | 0.002 | ** |
