# Supplementary material for: Parcel-guided rTMS for depression
Source: Transl Psychiatry. 2020 Aug 12;10:283. doi: 10.1038/s41398-020-00970-8 (PMC7423622; doi:10.1038/s41398-020-00970-8)
Supplement: Supplementary file 6 — Supplementary Table 4.2. [file 41398_2020_970_MOESM6_ESM.docx]

|  | **group** | **estimate** | **SE** | **t.ratio** | **p.value** | **sig** | **corrected.p** | **sig.corrected** |
| --- | --- | --- | --- | --- | --- | --- | --- | --- |
| **46 to s32** | sdTMS | -109.317 | 46.424 | -2.355 | 0.026 | * | 0.077 |  |
| **46 to s32** | pgTMS | 49.202 | 46.942 | 1.048 | 0.304 |  | 0.428 |  |
| **46 to ventral** | sdTMS | -28.242 | 31.649 | -0.892 | 0.380 |  | 0.428 |  |
| **46 to ventral** | pgTMS | 46.092 | 57.295 | 0.804 | 0.428 |  | 0.428 |  |
| **s32 to ventral** | sdTMS | -196.639 | 53.365 | -3.685 | 0.001 | *** | 0.006 | ** |
| **s32 to ventral** | pgTMS | 57.734 | 37.321 | 1.547 | 0.133 |  | 0.266 |  |
