# Supplementary material for: Parcel-guided rTMS for depression
Source: Transl Psychiatry. 2020 Aug 12;10:283. doi: 10.1038/s41398-020-00970-8 (PMC7423622; doi:10.1038/s41398-020-00970-8)
Supplement: Supplementary file 7 — Supplementary Table 5.1. [file 41398_2020_970_MOESM7_ESM.docx]

|  | **Estimate** | **Std..Error** | **df** | **t.value** | **Pr…t..** | **sig** | **corrected.p** | **sig.corrected** |
| --- | --- | --- | --- | --- | --- | --- | --- | --- |
| **46 to s32** | 0.138 | 0.041 | 32.742 | 3.345 | 0.002 | ** | 0.006 | ** |
| **46 to ventral** | 0.035 | 0.053 | 33.511 | 0.654 | 0.517 |  | 0.517 |  |
| **s32 to ventral** | 0.105 | 0.036 | 32.818 | 2.907 | 0.006 | ** | 0.010 | ** |
