# Supplementary material for: Parcel-guided rTMS for depression
Source: Transl Psychiatry. 2020 Aug 12;10:283. doi: 10.1038/s41398-020-00970-8 (PMC7423622; doi:10.1038/s41398-020-00970-8)
Supplement: Supplementary file 8 — Supplementary Table 5.2. [file 41398_2020_970_MOESM8_ESM.docx]

|  | **group** | **estimate** | **SE** | **df** | **t.ratio** | **p.value** | **sig** | **corrected.p** | **sig.corrected** |
| --- | --- | --- | --- | --- | --- | --- | --- | --- | --- |
| **46 to s32** | sdTMS | -0.076 | 0.022 | 33.130 | -3.425 | 0.002 | ** | 0.005 | ** |
| **46 to s32** | pgTMS | 0.062 | 0.035 | 33.130 | 1.780 | 0.084 |  | 0.168 |  |
| **46 to ventral** | sdTMS | 0.019 | 0.030 | 33.458 | 0.626 | 0.535 |  | 0.535 |  |
| **46 to ventral** | pgTMS | 0.054 | 0.044 | 33.458 | 1.227 | 0.228 |  | 0.312 |  |
| **s32 to ventral** | sdTMS | -0.070 | 0.019 | 33.075 | -3.603 | 0.001 | ** | 0.005 | ** |
| **s32 to ventral** | pgTMS | 0.035 | 0.030 | 33.075 | 1.147 | 0.260 |  | 0.312 |  |
