# Supplementary material for: Parcel-guided rTMS for depression
Source: Transl Psychiatry. 2020 Aug 12;10:283. doi: 10.1038/s41398-020-00970-8 (PMC7423622; doi:10.1038/s41398-020-00970-8)
Supplement: Supplementary file 9 — Supplementary Table 5.3. [file 41398_2020_970_MOESM9_ESM.docx]

|  | **Estimate** | **Std..Error** | **t.value** | **Pr…t..** | **sig** | **corrected.p** | **sig.corrected** |
| --- | --- | --- | --- | --- | --- | --- | --- |
| **46 to s32** | 142.001 | 93.913 | 1.512 | 0.142 |  | 0.158 |  |
| **46 to ventral** | 99.646 | 68.577 | 1.453 | 0.158 |  | 0.158 |  |
| **s32 to ventral** | 262.504 | 82.685 | 3.175 | 0.004 | ** | 0.011 | * |
