# Supplementary material for: Parcel-guided rTMS for depression
Source: Transl Psychiatry. 2020 Aug 12;10:283. doi: 10.1038/s41398-020-00970-8 (PMC7423622; doi:10.1038/s41398-020-00970-8)
Supplement: Supplementary file 10 — Supplementary Table 5.4. [file 41398_2020_970_MOESM10_ESM.docx]

|  | **group** | **estimate** | **SE** | **t.ratio** | **p.value** | **sig** | **corrected.p** | **sig.corrected** |
| --- | --- | --- | --- | --- | --- | --- | --- | --- |
| **46 to s32** | sdTMS | -109.317 | 47.221 | -2.315 | 0.028 | * | 0.085 |  |
| **46 to s32** | pgTMS | 49.202 | 46.942 | 1.048 | 0.304 |  | 0.364 |  |
| **46 to ventral** | sdTMS | -53.554 | 38.228 | -1.401 | 0.173 |  | 0.259 |  |
| **46 to ventral** | pgTMS | 46.092 | 57.295 | 0.804 | 0.428 |  | 0.428 |  |
| **s32 to ventral** | sdTMS | -196.639 | 54.317 | -3.620 | 0.001 | ** | 0.007 | ** |
| **s32 to ventral** | pgTMS | 57.734 | 37.321 | 1.547 | 0.133 |  | 0.259 |  |
