# Supplementary figures and images for: Parcel-guided rTMS for depression
Source: Transl Psychiatry. 2020 Aug 12;10:283. doi: 10.1038/s41398-020-00970-8 (PMC7423622; doi:10.1038/s41398-020-00970-8)

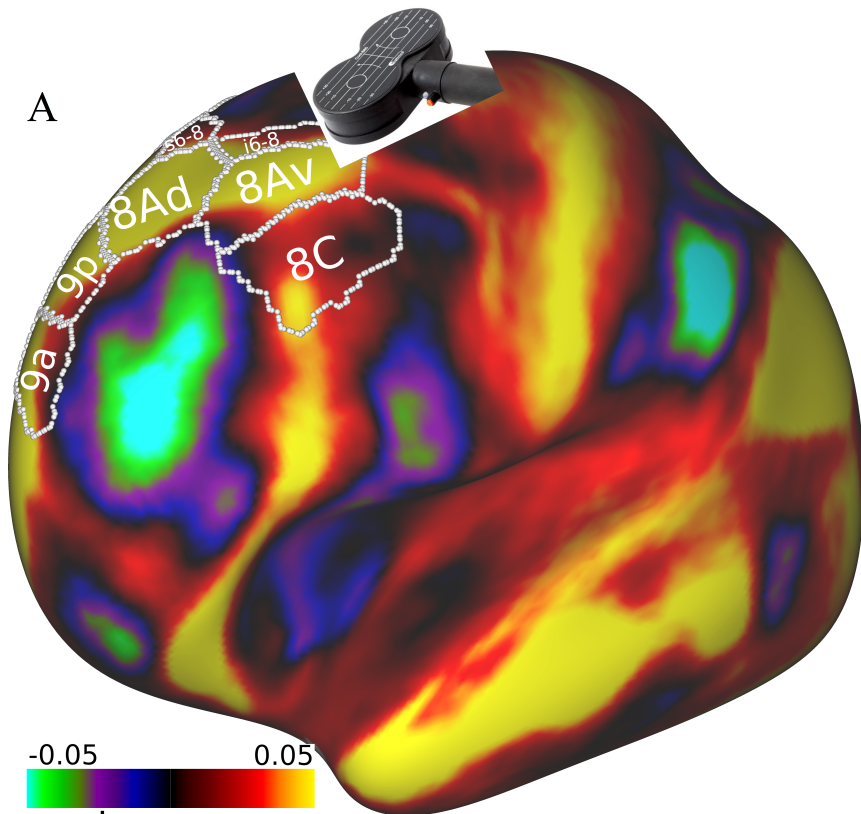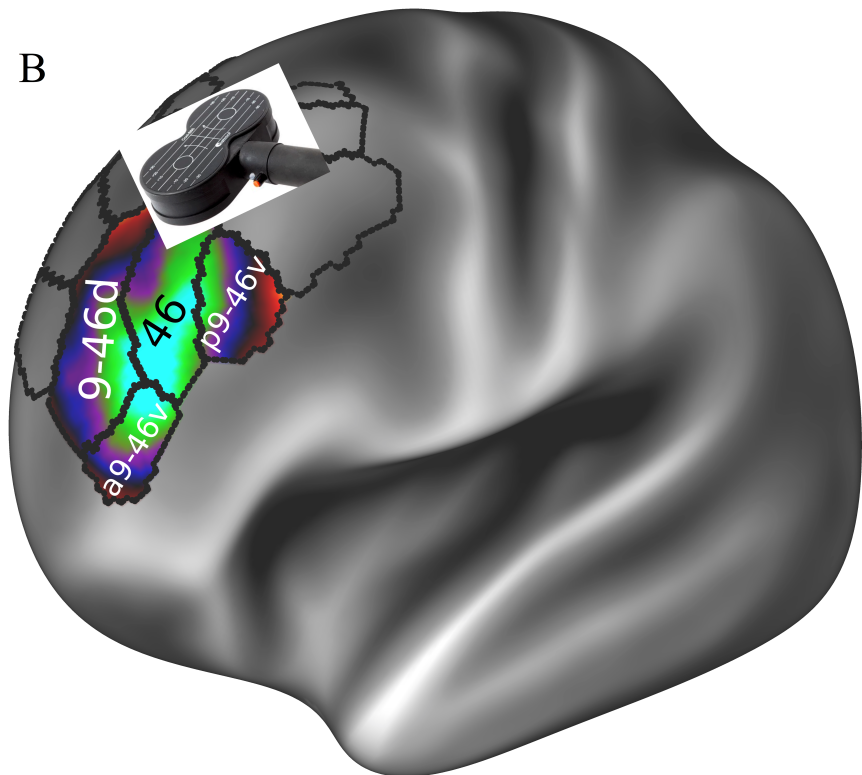

Supplement: Supplementary file 11 — Supplementary Figure 1. [file 41398_2020_970_MOESM11_ESM.pdf]

# 32 TRD Outpatients

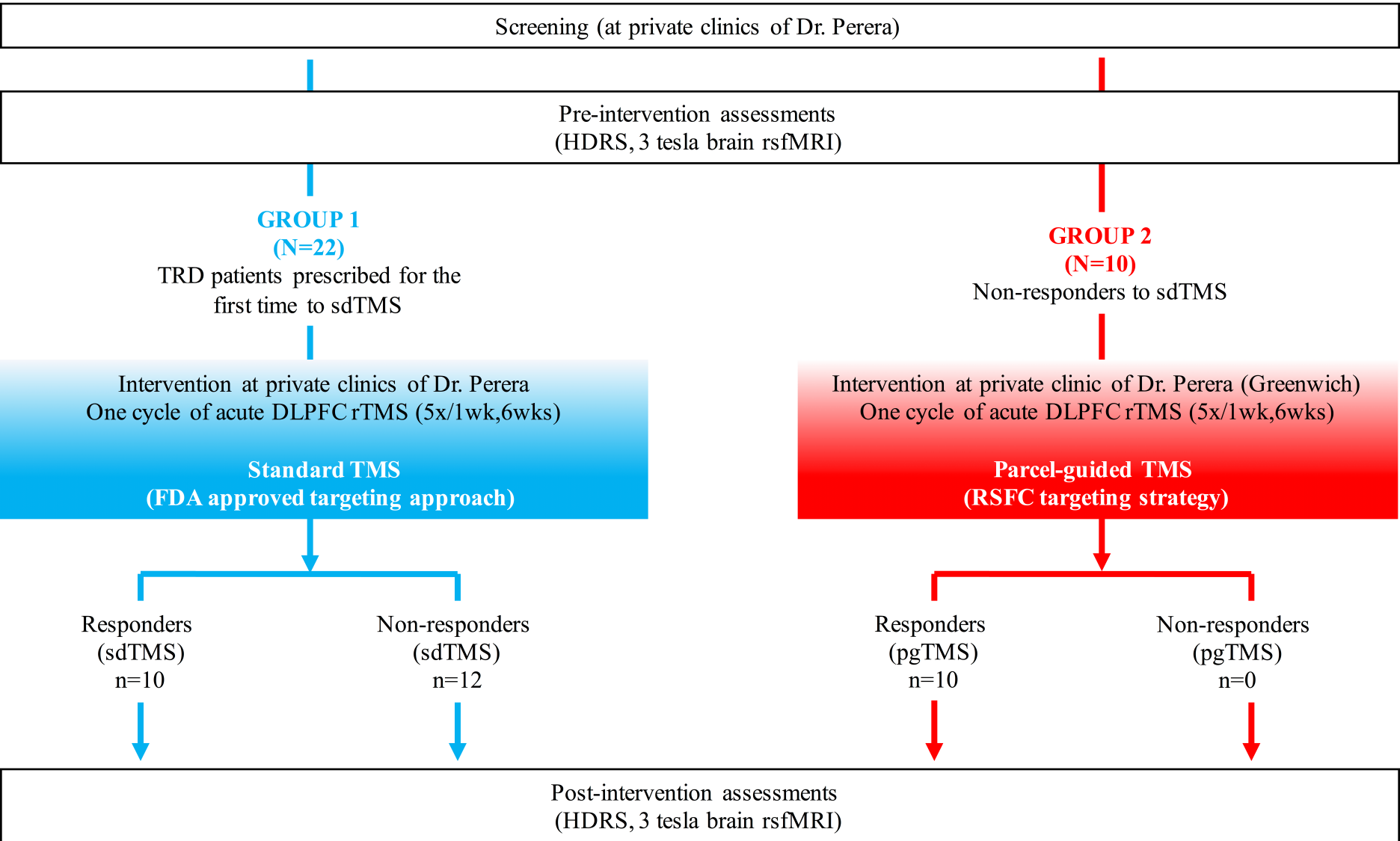

Supplement: Supplementary file 12 — Supplementary Figure 2. [file 41398_2020_970_MOESM12_ESM.pdf]

A

5-cm-rule  
(8Av)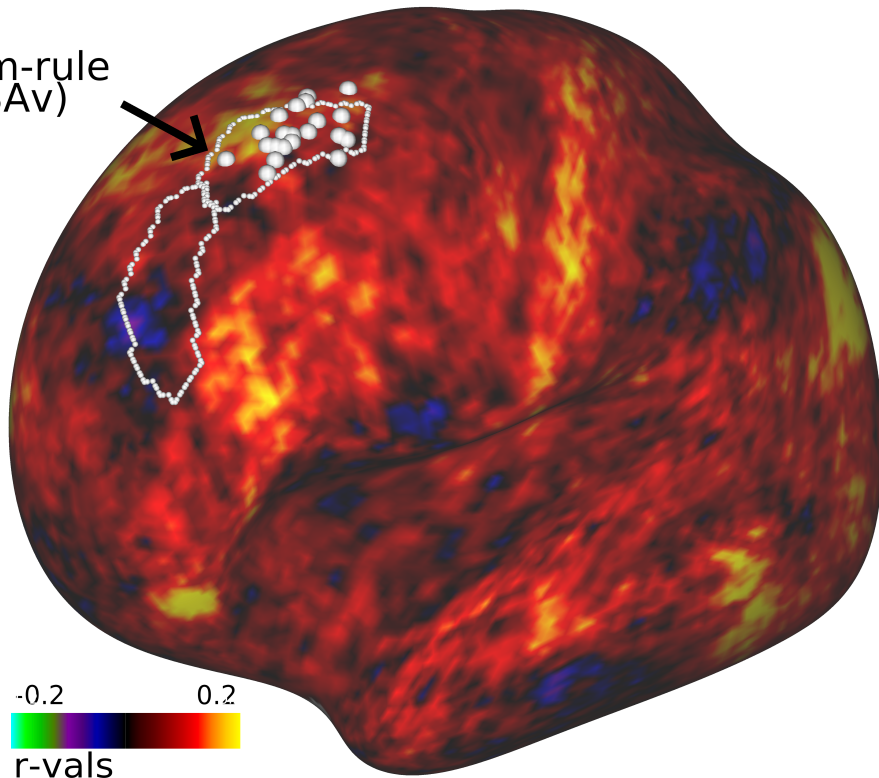

B

rsfc-guided  
(46)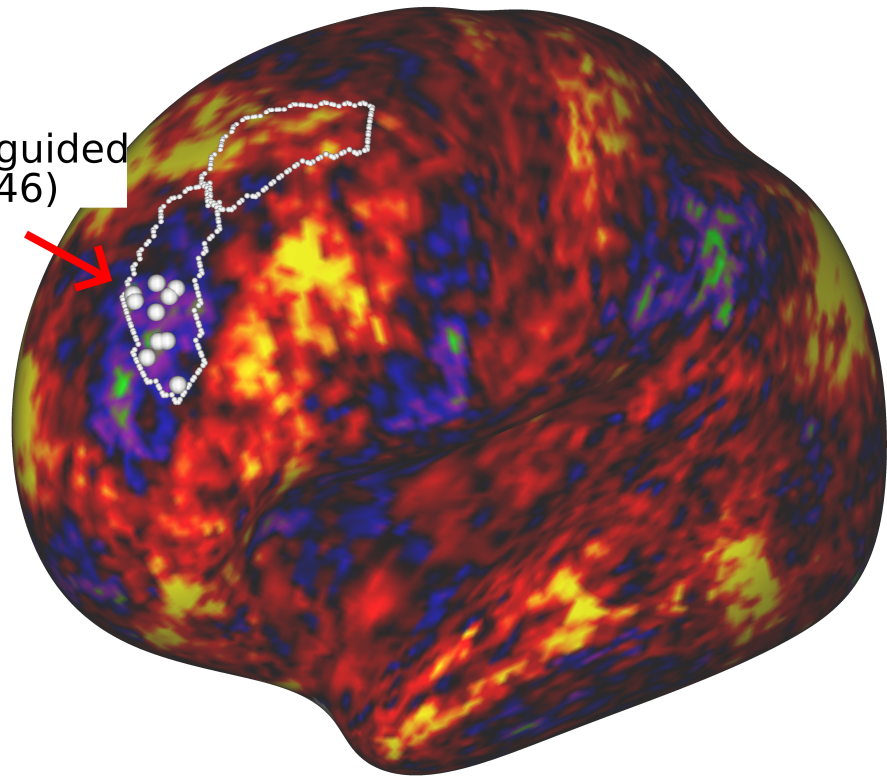

Supplement: Supplementary file 13 — Supplementary Figure 3. [file 41398_2020_970_MOESM13_ESM.pdf]
